# Taste Profile and Relative Bioavailability of Tovorafenib Powder for Oral Suspension and Food Effect of the Tovorafenib Tablet in Healthy Participants

**DOI:** 10.1002/cpdd.1558

**Published:** 2025-06-19

**Authors:** Yang Zhang, Mike Preigh, Jing Wang, Eleni Venetsanakos, Yujin Wang, Elly Barry, Don Corson

**Affiliations:** ^1^ Clinical Pharmacology Day One Biopharmaceuticals, Inc. Brisbane CA USA; ^2^ Chemistry, Manufacturing, and Controls Day One Biopharmaceuticals, Inc. Brisbane CA USA; ^3^ Translational Sciences Day One Biopharmaceuticals, Inc. Brisbane CA USA; ^4^ Clinical Development Day One Biopharmaceuticals, Inc. Brisbane CA USA

**Keywords:** food effect, pediatric, relative bioavailability, taste and palatability, type II kinase inhibitor

## Abstract

A pediatric‐friendly powder for oral suspension (PfOS) of tovorafenib, a type II RAF inhibitor, was developed for patients with difficulty swallowing tablets. This open‐label, randomized, phase 1 study (QSC205140) evaluated the taste/palatability of PfOS formulations (n = 12), the relative bioavailability of the PfOS versus tablet formulation, and the food effect on tablets (n = 12) in healthy participants. Tovorafenib was initially administered at 300 mg and reduced to 100 mg due to musculoskeletal adverse events (AEs). The addition of sweetener and/or flavoring improved taste/palatability. Geometric mean ratios (90% confidence interval) of dose‐corrected peak plasma drug concentration (C_max_/D) and area under the plasma concentration–time curve from time zero to the last measurable concentration (AUC_0‐last_/D) between the PfOS and tablet formulations were 96% (83%‐111%) and 104% (95%‐115%), respectively. Compared with fasted administration, administration of the tablet with food resulted in a 2‐3.5‐hours delay in time to C_max,_ and a 20% reduction in C_max_/D with no change in AUC_0‐last_/D. Four severe and 7 moderate AEs occurred with 300 mg of tovorafenib. All remaining AEs, reported with both 100 mg and 300 mg, were mild. These data suggest that tovorafenib PfOS and tablet formulations are comparable, and that the tablet can be administered with or without food.

Pediatric low‐grade glioma (pLGG) is the most common childhood central nervous system tumor and accounts for approximately 30% of pediatric brain tumors, with approximately 1000‐1600 anticipated new cases occurring annually in the United States.^1^, ^2^, ^3^ Alterations in v‐Raf murine sarcoma viral oncogene homolog B (*BRAF*) are present in up to 70% of pLGG cases.^4^, ^5^

Tovorafenib is an orally administered, selective, central nervous system‐penetrant, small‐molecule, type II RAF inhibitor with activity against monomeric (Class I alterations) and dimeric (Class II alterations, including fusions) forms of RAF signaling.^6^ Tovorafenib is also known as AMG‐2112819, BIIB‐024, BSK1369, MLN‐2480, TAK‐580, and DAY101, and its chemical name is 6‐amino‐5‐chloro‐N‐[(1R)‐1‐[5‐[[[5chloro‐4‐(trifluoromethyl)‐2‐pyridinyl]amino]carbonyl]‐2‐thiazolyl]ethyl]‐4‐pyrimidinecarboxamide.^6^, ^7^ In several clinical studies, tovorafenib exhibited evidence of antitumor activity in patients with tumors bearing either a *BRAF* fusion or a BRAF V600E mutation.^6^, ^8^, ^9^, ^10^, ^11^, ^12^ Tovorafenib is used for the treatment of patients aged 6 months and older with relapsed or refractory pLGG harboring a *BRAF* fusion or rearrangement, or a BRAF V600 mutation.^13^ In 76 evaluable patients with relapsed/refractory pLGG in the FIREFLY‐1 arm 1 (NCT04775485), tovorafenib (420 mg/m^2^ once weekly; 600 mg maximum) demonstrated an overall response rate of 51% by an independent radiology review committee based on Response Assessment in Pediatric Neuro‐Oncology low‐grade glioma criteria. Median duration of response was 13.8 months.^13^, ^14^ The Phase 3 LOGGIC/FIREFLY‐2 trial (NCT05566795) evaluating tovorafenib (380 mg/m^2^ once weekly; 600 mg maximum) in front‐line *BRAF*‐altered pLGG in comparison with standard of care chemotherapy is enrolling globally.^15^

Pharmacokinetics (PK) of tovorafenib have been studied in adult patients with advanced solid tumors.^12^ The peak plasma concentration and exposure of tovorafenib were roughly dose‐proportional over the dose ranges of 20‐280 mg every 2 days and 400‐800 mg every week, with a half‐life of approximately 70 hours (range 31‐119 hours), and a median time to the maximum observed plasma concentration (t_max_) of 3 hours (range 1‐24 hours) after dosing.^12^ In vitro studies suggest that tovorafenib is primarily metabolized by aldehyde oxidase and cytochrome P450 (CYP) 2C8, with minor contributions from CYP3A, CYP2C9, and CYP2C19.^6^, ^16^ A Phase 1 study of [^14^C]‐tovorafenib at a single 100‐mg oral dose in healthy male participants showed that tovorafenib was the most abundant circulating component in plasma, accounting for 78.7% of total plasma radioactivity exposure. Following a single oral dose of radiolabeled tovorafenib, 65% of the total radiolabeled dose was recovered in the feces (8.6% unchanged) and 27% of the dose was recovered in the urine (0.2% unchanged), suggesting that the major route of elimination is hepatic metabolism and biliary excretion.^6^, ^16^ In vitro studies have shown that tovorafenib both inhibits and induces certain CYP enzymes, including CYP2C8, CYP2C9, CYP2C19, and CYP3A at clinically relevant concentrations.^6^, ^16^ Tovorafenib also induces CYP1A2 and CYP2B6 and inhibits breast cancer resistance protein at clinically relevant concentrations.^6^, ^16^ Related drug–drug interaction clinical studies of tovorafenib are ongoing.

Tovorafenib was initially formulated as a tablet for clinical studies in adults. However, as enrollment in the pivotal FIREFLY‐1 trial was in a pediatric population (aged 6 months to 25 years), it was essential to develop a tovorafenib powder for oral suspension (PfOS) formulation to provide an alternate, easier to administer formulation for participants who are unable to swallow tablets. Furthermore, the PfOS could also provide more accurate dosing based on body surface area for younger patients compared with the administration of the tablet formulation.

This 2‐part, single‐center, open‐label, randomized Phase 1 study (QSC205140) aimed to evaluate and compare a novel PfOS of tovorafenib to the existing tablet.

## Methods

### Study Design

Part 1 of the study evaluated the taste and palatability attributes (smell, sweetness, bitterness, flavor, mouth feel/texture, and aftertaste) and overall acceptability of the tovorafenib PfOS with added sweeteners and flavors. The formulation with optimal flavor and sweetener level was used for further evaluation in Part 2 of the study. Taste and palatability in Part 2 of the study determined the relative bioavailability and evaluated the PK profiles of the preferred tovorafenib PfOS from Part 1 in comparison with the tablet and assessed the potential effects of food on the PK of tovorafenib following a single dose of the tablet in healthy participants in a fed state using a randomized, 3‐period crossover design. The study was conducted by Quotient Sciences–Miami, Inc. (Miami, FL) on behalf of Day One Biopharmaceuticals, Inc. The study protocol and consent form were approved by the Institutional Review Board (Advarra, Columbia, MD). All participants voluntarily signed the consent form before any study‐specific procedures were performed.

### Participants

Healthy male and female participants between 18 and 55 years of age with a body mass index of 18.0‐32.0 kg/m^2^ were included in the study. Eligible female participants included those who were not pregnant or lactating in Part 1 of the study and of non–childbearing potential in Part 2 of the study. Key exclusion criteria included clinically significant abnormal clinical chemistry, hematology, or urinalysis; history of clinically significant cardiovascular, renal, hepatic, chronic respiratory, or gastrointestinal disease (except cholecystectomy); neurological or psychiatric disorder, as judged by the investigator; significant serious skin disease, including rash, food allergy, eczema, psoriasis, or urticaria; and participants who were taking, or had taken, any prescribed or over‐the‐counter drug or herbal remedies (other than up to 4 g/day acetaminophen or hormone replacement therapy; hormonal contraception was permitted in Part 1 only) in the 14 days before tovorafenib administration.

### Taste and Palatability Ass essments

In Part 1 of the study, Formulation A was the reference and contained no sweetener or flavoring, Formulation B contained only sweetener (sucralose), and Formulations C through F contained strawberry or vanilla flavoring with differing levels of sweetener (sucralose) (Table 1). All formulations were reconstituted to 25 mg/mL of tovorafenib.

**Table 1:** Summary of PfOS Formulations

Formulations	Flavoring/Sweetener	Tovorafenib
A	Reference, no flavor or sweetener	25 mg/mL
B	No flavor/0.15% sucralose	
C	0.4% Strawberry/0.15% sucralose	
D	0.4% Strawberry/0.3% sucralose	
E	0.4% Vanilla/0.3% sucralose	
F	0.4% Vanilla/0.15% sucralose	

PfOS, powder for oral suspension.

Twelve participants received training in the “sip and spit” technique on the day before administration of one of the PfOS formulations and completed a practice questionnaire. On the study day, participants were randomized to 1 of 6 sequences (ABFCED, BCADFE, CDBEAF, DECFBA, EFDACB, and FAEBDC, with 2 participants assigned to each sequence) (Figure S1A). Participants received 5 mL of a particular PfOS and were instructed to hold it in their mouths for approximately 30 seconds before expectoration. Participants were instructed not to swallow any of the PfOS. There was a 1‐hour minimum washout between tasting each formulation. During this time, participants cleansed their palates using mineral water and unsalted crackers. Taste and palatability were assessed using a questionnaire in which participants rated the overall acceptability of each formulation (independent of any previous formulations) on specific palatability attributes (smell, sweetness, bitterness, flavor, mouth feel/texture, and aftertaste) on a 9‐point scale (1 = dislike extremely; 2 = dislike very much; 3 = dislike moderately; 4 = dislike slightly; 5 = neither dislike nor like; 6 = like slightly; 7 = like moderately; 8 = like very much; or 9 = like extremely) independent of any previous formulations. Preferences for a particular flavored formulation were grouped as follows: Grades 1‐3 = dislike; Grades 4‐6 = neutral; Grades 7‐9 = like. This 9‐point hedonic scale was developed and validated by Peryam et al.^17^ as a method of measuring food preference and has been used in the assessment of taste/palatability for pharmaceutical products.^18^, ^19^ The rating scale used is provided in Figure S2. Each participant had up to 15 minutes to complete the questionnaire individually and in private before the next PfOS tasting.

### Relative Bioavailability and Food ‐ Effect A ssessments

In Part 2 of the study, 12 participants were randomized to 1 of 3 sequences (T[fast]S[fast]T[fed], S[fast]T[fed]T[fast], and T[fed]T[fast]S[fast]) (Figure S1B). In each treatment period, tovorafenib was administered on Day 1 as a tablet under fasted (T[fast]), as PfOS under fasted (S[fast]), or as a tablet after a high‐fat meal (T[fed]). Tovorafenib was administered in the morning after an overnight fast of 10 hours or more. For treatment groups T[fast] and S[fast], tovorafenib was given under fasted conditions (ie, no breakfast), while for the T[fed] group, it was administered 30 minutes after the start of a high‐fat meal. A total of 240 mL of water was administered with each tovorafenib tablet. For the PfOS, immediately after administration, the dosing vessel was rinsed with water, and participants were required to consume the rinse solution and additional water up to a total volume of 240 mL (including the dosing volume and volume used to rinse the dosing vessel). If needed, additional water in 50‐mL aliquots was provided and recorded but was not classed as a protocol deviation. Participants were required to fast for 4 hours or more after single‐dose administration of the tovorafenib tablet. The composition of the high‐fat meal consisted of 800‐1000 calories, of which approximately 54% of calories were from fat. To be eligible for dosing, participants were required to consume at least 90% of the meal provided. Intake of fluids was prohibited 1 hour before dosing until 1 hour after dosing, except for fluid consumed with breakfast before dosing and water for tovorafenib tablet intake. Blood samples were collected at regular intervals (see Blood Collection and Processing for PK Analysis in the Pharmacokinetic Assessments section for more details) for PK analysis from before dosing to 120 hours after dosing. Participants were required to remain in the clinical unit until 96 hours after dosing (Day 5) and to return to the clinic at 120 hours after dosing (Day 6) so a final blood sample could be taken for PK analysis. There was a minimum 14‐day washout between each dose. A follow‐up call took place 7‐10 days after the final dose to ensure the ongoing well‐being of the participants. Participants could withdraw from the study at any time for any reason.

In Period 1, participants received 300 mg of tovorafenib in tablet form (3 × 100 mg tovorafenib tablets) or PfOS in a fasted state, or only a tablet after a high‐fat meal. The highest dose strength for a tovorafenib tablet is 100 mg. All 12 participants experienced nonserious musculoskeletal adverse events (AEs) following administration of tovorafenib at this dose; thus, for Periods 2 and 3, the dose was reduced to 100 mg. Participants were also premedicated with ibuprofen to mitigate any musculoskeletal AEs. An initial dose of 400 mg of ibuprofen was given between 30 minutes and 1 hours before tovorafenib dosing and then approximately every 8 hours (± 1 hour) after dosing, up to 48 hours after dosing. Beyond 48 hours, participants could continue taking ibuprofen as needed.

### Pharmacokinetic Assessments

#### Blood Collection and Processing for PK Analysis

During each treatment period, serial blood samples were collected before dosing and up to 120 hours after dosing at the following time points for the determination of tovorafenib plasma concentrations: 0 hour before dosing and 0.5, 1, 1.5, 2, 3, 4, 5, 6, 8, 10, 12, 16, 24, 40, 48, 60, 72, 96, and 120 hours after dosing. Blood samples were collected in dipotassium ethylenediaminetetraacetic acid tubes and centrifuged within 30 minutes of collection. Following centrifugation, aliquots of harvested plasma were transferred into cryovials and stored at −70°C until the time for sample analysis.

Plasma concentrations of tovorafenib were determined by a validated liquid chromatography with tandem mass spectrometry–based method with a lower limit of quantification of 0.5 ng/mL.

Following sample thawing, a 50‐µL plasma aliquot was added to a 96‐well plate, followed by adding 50 µL of isotope‐labeled internal standard (5x^13^C‐tovorafenib) solution. Plasma proteins were precipitated by the addition of 300 µL of acetonitrile:formic acid solution at 100:1 (v:v). The plate was covered, vortexed, and centrifuged for approximately 10 minutes at 3000 rpm before transfer of about 300 µL of supernatant into another clean 96‐well plate. The supernatant was dried down completely, then 200 µL of reconstitution solvent (acetonitrile:water at 50:50 [v:v]) was added into a 96‐well plate and vortex mixed again before sample injection into a reversed‐phase high‐performance liquid chromatography with a Turbo Ion Spray tandem mass spectrometry system.

A reverse‐phase gradient method running at a flow rate of 0.40 mL/min on a Venusil C18 5 µm column, 2.1 × 50 mm (Phenomenex) provided tovorafenib retention time at approximately 1.70 minutes. The mobile phases used were water:formic acid at 100:0.1 (v:v) (A); and acetonitrile:water:formic acid at 95:5:0.1 (v:v:v) (B) under a positive ion spray mode and detected through multiple reaction monitoring of mass transition pairs at m/z 506.2→334.2 and 511.2→334.2 for tovorafenib and its internal standard, respectively.

A calibration curve was established using the peak area ratios of the analyte against its isotopically labeled internal standard. Linearity with 1/x^2^ weighted least‐squares regression was achieved in the tovorafenib concentration range of 0.500‐3500 ng/mL with quality control samples ranging from 1.5 to 2800.0 ng/mL. The results for intraday precision and accuracy were 0.6%‐12.6% and −4.4% to 3.6%, respectively; interday precision and accuracy were 1.9%‐9.5% and −1.3 to 1.4%, respectively. Incurred sample reanalysis was conducted in about 10% of study samples, and results met acceptance criteria.

### Pharmacokinetic A nalysis

PK parameters assessed included area under the plasma concentration–time curve (AUC) from time zero to the time of last measurable concentration (AUC_0‐last_), AUC from time zero to infinity (AUC_0‐inf_), maximum observed plasma concentration after drug administration (C_max_), time to C_max_ (t_max_), and the elimination half‐life associated with the terminal slope of a semilogarithmic concentration–time curve (t_1/2_).

### Safety Assessments

Safety evaluations included analysis of AEs, laboratory variables (hematology, clinical chemistry, and urinalysis), vital signs, electrocardiogram, and a physical examination. AEs were coded using the Medical Dictionary for Regulatory Activities Version 24.0. The severity of AEs was assessed as follows: “mild” was defined as an AE that was easily tolerated by the participant, caused minimal discomfort, and did not interfere with everyday activities; “moderate” was defined as an AE that was sufficiently discomforting to interfere with normal everyday activities, and intervention may have been needed; and “severe” was defined as an AE that prevented normal everyday activities and treatment, or other intervention, was needed. Treatment‐emergent AEs (TEAEs) were summarized by frequency, severity, and relatedness to the study drug. TEAE frequency (the number of TEAEs and the number of participants experiencing a TEAE) was tabulated by system organ class and preferred term. In the per‐participant analyses, a participant having the same event more than once was counted only once. Adverse drug reactions were defined as any AE where a causal relationship with the study drug was at least a reasonable possibility, that is, “related.” Serious AEs were deemed as any AE that resulted in death, was life‐threatening or required hospitalization, resulted in persistent or significant disability/incapacity, or was an important medical event as recognized by the investigator.

### Statistical Analysis

For Part 1 of the study, evaluable participants were defined as those participants who had completed the taste/palatability questionnaires for all formulations. Each taste/palatability attribute and overall acceptability were compared among the 6 formulations using the nonparametric Friedman test at a 5% significance level. The null hypothesis was that there was no statistically significant difference between the formulations in each taste/palatability attribute or overall acceptability. If the Friedman test showed significance at the 5% level, pairwise comparisons were conducted using Wilcoxon signed‐rank tests, and a *P* value was assigned for each test formulation (Formulations B through F) versus the reference (Formulation A) for each attribute and overall acceptability. Due to the exploratory nature of the study, there were no adjustments for multiple comparisons, and *P* values are presented for descriptive purposes only.

For Part 2 of the study, plasma PK parameters for tovorafenib were calculated from the individual plasma concentration profiles by noncompartmental analysis methods, applying the linear trapezoidal methods, implemented in Phoenix WinNonlin (Version 8.0, Certara USA, Inc.). Descriptive statistics (mean, standard deviation, geometric mean ratio [GMR], and geometric coefficients of variation) were calculated for all PK parameters except for t_max_, for which only the median and range were determined. A linear mixed‐effects model was fitted to the log‐transformed PK parameters (AUC_0‐last_, AUC_0‐inf_, and C_max_). The model included treatment, period, and sequence as fixed factors and participants nested within sequence as a random factor. A formal statistical analysis was performed on the dose‐corrected PK parameters C_max_, AUC_0‐last_, and AUC_0‐inf_ to assess the relative bioavailability and the presence of a food effect on tovorafenib. To evaluate the relative bioavailability of tovorafenib PfOS (fasted) compared with tovorafenib tablet (fasted), and the food effect on tovorafenib tablet (fed compared with fasted), a point estimate and the corresponding 90% confidence interval for the difference between least‐square means of the test (PfOS, fasted; or tablet, fed) and reference (tablet, fasted) were calculated. These were back‐transformed to obtain the point estimate and the 90% confidence interval for GMR on the untransformed scale. The PK analysis set, defined for Part 2 only, included all participants who received at least 1 dose of the study drug and who satisfied the following criteria for at least 1 profile: no missing samples or invalid postdose analytical results at critical time points (eg, around C_max_); no relevant protocol deviations that may impact the study objectives with respect to the PK end points; no relevant AEs such as vomiting that suggest that the whole dose was not available for absorption for a particular participant. The PK analysis subset, defined for Part 2 only, included evaluable participants who received the T[fast] and S[fast] regimens and who had PK data up to 72 hours after dosing for the relative bioavailability assessment, and evaluable participants who had received the T[fast] and T[fed] regimens and who had PK data up to 72 hours after dosing for the food‐effect assessment. PK parameters were excluded from the summary statistics if measurable predose values were observed at greater than 5% of C_max_.

Nonmeasurable values reported in the plasma concentration data (ie, values that are below the limit of quantitation [BLQ]) were set to zero for the determination of summary statistics, except for geometric means, geometric standard deviation, and geometric coefficient of variation, where BLQ values were imputed as half the lower limit of quantification value. For all plots on a linear scale, concentration values reported as BLQ were presented as zero.

No formal sample size calculation was made, as the study was exploratory. Twelve participants were enrolled for each part of the study to ensure a minimum of 10 evaluable participants, which was considered appropriate to meet the objectives of the study.

## Results

### Study Population

In Part 1 of the study, 12 participants were randomized to 1 of the 6 sequences and completed the study. All 12 participants received at least 1 dose of tovorafenib and were included in the safety and taste analysis sets. In Part 2 of the study, 12 participants were enrolled and randomized to 1 of the 3 sequences. All 12 participants received at least 1 dose of tovorafenib, had a minimum of 1 valid postdose analytical result for PK parameter estimation, and satisfied the criteria for at least 1 profile. All 12 participants were therefore included in the safety and PK analysis sets. One participant did not receive reference formulation (T[fast]) and was therefore excluded from the PK analysis subset for the formal statistical analysis. A summary of the participant and baseline characteristics is presented in Table S1. In both Parts 1 and 2, the majority of participants were White (91.7% and 83.3%, respectively), with an equal distribution of women and men. The mean age was 40.3 (range, 36‐52) years in Part 1 and 42.5 (range, 35‐54) years in Part 2. Mean body mass index was 26.4 kg/m^2^ in Part 1 and 27.6 kg/m^2^ in Part 2.

### Taste and Palatability Attribute Evaluation

Median scores for each taste/palatability attribute (eg, smell, sweetness, bitterness, flavor, mouth feel/texture, and aftertaste) and overall acceptability are summarized in Table 2. The lowest median score, 3.0, was observed in the reference formulation (Formulation A) for flavor, mouth feel/texture, aftertaste, and overall acceptability. Addition of a sweetener (Formulation B) improved median scores for all these 3 attributes, as well as the overall acceptability. Flavoring paired with different amounts of sweetener (Formulation C through F) provided a more robust improvement over the reference product for overall acceptability and each taste attribute except bitterness, as indicated by further increased median scores. There did not appear to be a clear preference for a particular flavored formulation. The formulation with the highest proportion scored as “like” was Formulation C (0.4% strawberry flavoring and 0.15% sucralose).

**Table 2:** Taste/Palatability Attributes Scores and Assessment for Tovorafenib PfOS Formulations (Test Treatment 0.4% Flavoring/Sucralose %)

Formulations	A (NA/NA)	B (NA/0.15%)		C (Strawberry/0.15%)		D (Strawberry/0.3%)		E (Vanilla/0.3%)		F (Vanilla/0.15%)	
Taste attribute	Median score (minimum–maximum)	Median score (minimum–maximum)	Difference (*P* value)^a^	Median score (minimum–maximum)	Difference (*P* value)^a^	Median score (minimum–maximum)	Difference (*P* value)^a^	Median score (minimum–maximum)	Difference (*P* value)^a^	Median score (minimum–maximum)	Difference (*P* value)^a^
Smell	5.0 (1‐6)	5.0 (4‐7)	0.5 (.5)	7.0 (3‐8)	3.0 (.031)	7.0 (6‐8)	3.0 (.031)	7.0 (6‐8)	2.5 (.031)	7.0 (3‐9)	3.0 (.19)
Sweetness	4.0 (1‐6)	6.0 (1‐7)	2.0 (.094)	7.0 (4‐8)	3.5 (.004)	7.0 (3‐8)	2.0 (.004)	7.0 (4‐8)	3.0 (.002)	6.0 (3‐9)	2.0 (.047)
Bitterness^b^	4.0 (1‐7)	5.0 (3‐7)	NA	5.5 (4‐7)	NA	5.0 (4‐8)	NA	5.5 (5‐7)	NA	5.0 (2‐7)	NA
Flavor	3.0 (1‐6)	5.5 (3‐7)	2.0 (.004)	7.0 (3‐8)	3.0 (.002)	6.5 (3‐8)	3.0 (.004)	6.5 (5‐8)	4.0 (.004)	5.5 (3‐8)	3.0 (.016)
Mouth feel/texture	3.0 (1‐7)	4.0 (1‐7)	0.5 (.055)	6.0 (1‐8)	2.0 (.025)	5.5 (2‐8)	2.0 (.008)	6.0 (2‐8)	2.0 (.08)	5.0 (2‐8)	1.5 (.004)
Aftertaste	3.0 (1‐6)	4.0 (1‐7)	3.0 (.037)	6.0 (2‐8)	3.0 (.021)	7.0 (4‐8)	3.0 (.012)	7.0 (2‐8)	3.0 (.006)	5.0 (3‐7)	2.5 (.012)
**Overall**	**3.0 (1**‐**6)**	**5.0 (1**‐**7)**	**1.0 (.066)**	**7.0 (3**‐**8)**	**2.0 (<.001)**	**6.0 (3**‐**8)**	**3.0 (<.001)**	**6.5 (4**‐**8)**	**3.0 (.002)**	**5.5 (3**‐**8)**	**1.5 (.006)**

NA, not applicable; PfOS, powder for oral suspension.

Key for median score grade: 1 = dislike extremely, 2 = dislike very much, 3 = dislike moderately, 4 = dislike slightly, 5 = neither like nor dislike, 6 = like slightly, 7 = like moderately, 8 = like very much, 9 = like extremely. Participants tasted 5 mL of 6 different PfOS formulations (A‐F). Formulation A = 25 mg/mL tovorafenib with no sweetener/flavoring; Formulations B through F = 25 mg/mL tovorafenib with various levels of flavoring and sucralose.

^a^Median of the paired difference between test formulation versus reference formulation A, and *P* values from the Wilcoxon signed‐rank test. Due to the exploratory nature of the study, there were no adjustments for multiple comparisons, and *P* values are descriptive.

^b^ For the bitterness attribute, the *P* values were >0.05 from Friedman's test; therefore, no Wilcoxon signed‐rank test was conducted.

Friedman's test was statistically significant for overall acceptability and taste aspects such as smell, sweetness, flavor, mouth feel/texture, and aftertaste, indicating that at least 1 formulation has a significantly different taste score from the other formulations (*P* < .05). For bitterness, the *P* value was greater than .05, suggesting there was no significant difference between at least 2 of the regimens for bitterness scores. The pairwise comparisons were further conducted using the Wilcoxon signed‐rank test (Table 2). For all pairwise comparisons, the median of the paired differences was positive, indicating that each flavor/sweetener combination showed an improved acceptability for each taste aspect analyzed and overall acceptability when compared with the reference Formulation A with no sweetener or flavoring. The smallest improvements from Formulation A were observed with Formulation B (0.15% sucralose) across the taste aspects, except for aftertaste, where the improvement equaled those regimens with flavoring. Formulation C (strawberry flavor and 0.15% sucralose), Formulation D (strawberry flavor and 0.3% sucralose), and Formulation E (vanilla flavor and 0.3% sucralose), all demonstrated similar improvements across the taste aspects and overall acceptability when compared with reference Formulation A, with these improvements generally greater than Formulation F (vanilla flavor and 0.15% sucralose).

Overall, taste scores were significantly improved for the tovorafenib PfOS formulations with the addition of sweetener (sucralose) alone or additionally including strawberry or vanilla flavors, with greater improvement seen with the addition of both sweetener and flavors for overall acceptability and all taste aspects. Formulation C (0.4% strawberry flavor and 0.15% sucralose) was chosen for the relative bioavailability assessment in Part 2 based on the overall taste and palatability results from Part 1.

### Relative Bioavailability and Food Effect

**Figure 1:**
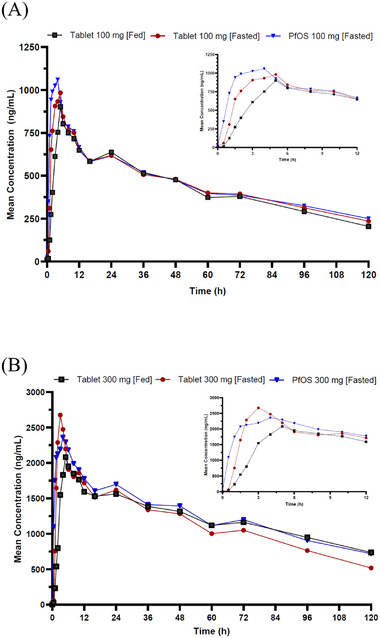
Mean plasma concentration–time profiles for tovorafenib at 100 mg and 300 mg under different prandial states and formulations (tablet vs. PfOS). Values below the limit of quantification were assigned a value of zero and were included in the calculation of the mean values. (A) Tovorafenib 100 mg and (B) tovorafenib 300 mg. PfOS, powder for oral suspension.

The mean plasma concentration–time profiles of tovorafenib across all 3 regimens (T[fast], S[fast], and T[fed]) at both 100‐mg and 300‐mg doses are presented in Figure 1A,1B, respectively.

A summary of PK parameters with a single dose of 100 or 300 mg of tovorafenib across all 3 regimens is provided in Table 3. In the fasted condition, similar exposure (AUC_0‐last_ and C_max_) was observed with both tablet and PfOS formulations at both 100‐ and 300‐mg doses. Administration of tovorafenib tablet with a high‐fat meal led to a delayed absorption, where the median t_max_ was 1.5 and 3.5 hours longer compared with the fasted condition at 100 and 300 mg, respectively. Additionally, the C_max_ values were slightly higher under fasted conditions than fed. However, the overall exposure AUC_0‐last_ was similar between fasted and fed states, suggesting food did not affect the extent of absorption.

**Table 3:** Summary of PK Parameters With 1 Dose of 100‐ and 300‐mg Tovorafenib

Dose/Formulation (regimen)	100‐mg tablet T[fast]	100‐mg PfOS S[fast]	100‐mg tablet T[fed]	300‐mg tablet T[fast]	300‐mg PfOS S[fast]	300‐mg tablet T[fed]
Number of participants	n = 6	n = 7	n = 8	n = 4	n = 4	n = 4
t_max_ (hours) Median (range)	3.50 (2.00‐5.00)	3.00 (1.50‐4.00)	5.00 (4.00‐24.0)	3.00 (3.00‐3.00)	3.00 (1.50‐4.00)	6.50 (4.00‐24.0)
C_max_ (ng/mL) Mean (SD) Geometric mean (% CV)	1210 (277) 1180 (21.6)	1140 (263) 1110 (22.0)	931 (219) 910 (23.4)	2680 (629) 2620 (24.3)	2490 (305) 2470 (11.7)	2230 (573) 2170 (26.5)
AUC_0‐last_, ng•h/mL						
Mean (SD)	54,000 (8130)	53,600 (12,400)	51,300 (9960)	138,000 (39,400)	153,000 (27,800)	144,000 (16,100)
Geometric mean (% CV)	53,500 (15.0)	52,400 (22.3)	50,400 (20.7)	133,000 (29.4)	151,000 (17.9)	143,000 (11.4)
t_1/2_ (hours)						
Mean (SD)	75.7 (21.1)	65.8 (8.83)	62.2 (8.63)	68.1 (11.7)	84.7 (20.0)	89.5 (33.5)
Geometric mean (% CV)	73.5 (26.0)	65.4 (13.2) (n = 6)^a^	61.7 (13.6) (n = 7)^a^	67.4 (17.2)	83.2 (22.0)	85.4 (39.2) (n = 3)^a^

AUC_0‐last_, area under the plasma concentration–time curve from time zero to the time of last measurable concentration; C_max_, maximum observed plasma concentration; CV, coefficient of variation; PfOS, powder for oral suspension; PK, pharmacokinetics; SD, standard deviation; S[fast], PfOS under fasted; t_1/2_, elimination half‐life associated with the terminal slope of a semilogarithmic concentration–time curve; t_max_, time to maximum observed plasma concentration; T[fast], tablet under fasted; T[fed], tablet after a high‐fat meal.

a PK parameters were excluded from the summary statistics due to an adjusted R^2^ of <0.9. The mean (arithmetic and geometric) PK parameters for plasma tovorafenib following single oral doses (100 mg and 300 mg) given to study participants in tablet or PfOS formulations in Part 2 of the study.

In the current study, the C_max_ and AUC_0‐last_ at 300 mg were approximately 2.3‐ and 2.5‐fold of that observed at 100 mg, respectively. Thus, a statistical comparison of tovorafenib dose‐corrected PK parameters (C_max_ and AUC_0‐last_) was further performed to assess the relative bioavailability and the food effect. A summary of the GMRs of C_max_/Dose (D) and AUC_0‐last_/D between the PfOS test (S[fast]) and the tablet reference formulation (T[fast]), along with systemic exposure parameters under fed and fasted conditions, is provided in Table 4.

**Table 4:** Dose‐Corrected PK Parameters for Tovorafenib Assessment of Relative Bioavailability and Food Effect

		Test		Reference			
Assessments	Parameters	n	Adj. geo mean	n	Adj. geo mean	Ratio^a^ (%)	90% CI
Relative bioavailability (PfOS/tablet)^b^	C_max_/D (ng/mL/mg)	10^d^	10.3	10^d^	10.7	96.0	(83.0‐111)
	AUC_0‐last_/D (ng•h/mL/mg)	10^d^	529	10^d^	508	104	(94.6‐115)
Food effect on tablet (fed/fasted)^c^	C_max_/D (ng/mL/mg)	11	8.43	10^d^	10.7	78.8	(68.3‐91)
	AUC_0‐last_/D (ng•h/mL/mg)	11	594	10^d^	508	97.3	(88.6‐107)

Adj. geo mean, adjusted geometric mean from model where pharmacokinetic parameters (ie, C_max_ and AUC) were adjusted for dose level; AUC_0‐last_, area under the plasma concentration–time curve from time zero to the time of last measurable concentration; CI, confidence interval; C_max_, maximum observed plasma concentration; D, dose; PfOS, powder for oral suspension; PK, pharmacokinetic.

^a^ Ratio of adjusted geometric mean for test: reference.

^b^PfOS: tovorafenib PfOS formulation, fasted (test); tovorafenib tablet formulation, fasted (reference).

^c^Fed: tovorafenib tablet formulation (test); fasted: tovorafenib tablet formulation (reference).

^d^PK parameters from 1 participant in the treatment group were excluded from the summary statistics due to measurable predose values observed at >5% of C_max_.

### Safety and Tolerability

There were no TEAEs reported during Part 1 of the study. In Part 2, 12 participants (100%) reported a total of 71 TEAEs, 60 of which were related to tovorafenib (Table S2). There was a higher incidence of adverse drug reactions reported in the 300‐mg dose regimens compared with the 100‐mg dose regimens. All 4 severe AEs (including myalgia and back pain) and 7 moderate AEs (including myalgia, pain in extremity, salivary gland pain, hypoesthesia, and paresthesia) were reported in the 300‐mg dose regimens and had resolved by the end of the study. All AEs in the 100‐mg dose regimens were mild. There were no serious AEs or AEs leading to study drug discontinuation in Part 2.

There were no clinically significant abnormal laboratory, vital sign, electrocardiogram, or physical examination findings noted in either part of the study.

## Discussion

The need for pediatric‐friendly formulations has been regularly reported by health care professionals, regulatory authorities, and international organizations, including the World Health Organization.^20^, ^21^, ^22^ Liquid medications enable more accurate weight‐based dosing, allow easier dose adjustments and administration, and can improve adherence to treatment and, in turn, therapeutic success.^22^, ^23^, ^24^ This need for pediatric‐friendly formulations is particularly the case in the pLGG setting, where chronic administration is often required with targeted therapies, and highlights the importance of this study evaluating tovorafenib PfOS formulations.

This study assessed the taste and palatability of 6 different tovorafenib PfOS formulations, and Formulation C (4% strawberry flavor with 0.15% sucralose) was deemed to have the most favorable palatability and overall acceptability. The relative bioavailability of Formulation C compared with the tablet was determined. Administration of both under fasted conditions resulted in similar exposure, indicating that the 2 formulations were comparable. Taken together, the results support the tovorafenib PfOS as a viable option for the targeted pediatric population.

The potential effect of food on the PK profiles of tovorafenib following a single dose of the tablet in the fed state was also assessed. Administration of the tovorafenib tablet with a high‐fat meal led to an approximate 20% decrease in peak exposure, without affecting overall exposure, suggesting that tovorafenib may be taken with or without food. This flexibility in taking tovorafenib without regard to food will provide more convenience for patients and caregivers and may improve treatment adherence.

Previous clinical studies of tovorafenib (given once weekly at 600 mg), in patients with solid tumors, showed that systemic exposure increased in a dose‐proportional manner between 20 and 280 mg every 2 days and 400 to 800 mg every week.^12^ This study initially started with the 300‐mg single dose to adequately characterize the PK of tovorafenib in healthy participants. However, nonserious musculoskeletal AEs (mild in 5, moderate in 3, and severe in 4) were observed in participants who received 300 mg of tovorafenib, with myalgia the most frequently reported AE (10 [83.3%] participants reporting 10 events). The 300‐mg dose was reduced to 100 mg, and prophylactic administration of ibuprofen was given to all participants in the subsequent periods of the study. After implementation of these measures, all musculoskeletal AEs reported in the remainder of the study were mild in severity. Similar musculoskeletal AEs were observed and reported in both Phase 1 and Phase 2 adult and pediatric studies of tovorafenib.^11^, ^12^, ^14^ The mechanism underlying tovorafenib‐related musculoskeletal AEs has not been studied. However, similar musculoskeletal AEs have been reported with BRAF inhibitors such as encorafenib, vemurafenib, and dabrafenib, whether used as monotherapy or in combination with mitogen‐activated protein kinase inhibitors, suggesting a potential class effect.^25^, ^26^, ^27^ Moreover, an inflammatory response may be implicated, as subjects with nonserious musculoskeletal AEs in this study responded well to nonsteroidal anti‐inflammatory drug treatment. This is consistent with earlier findings where inflammatory side effects, including arthralgias and myalgias, of BRAF and MEK inhibitors were described.^28^

Mean t_1/2_ values obtained from the 3 tovorafenib treatment regimens ranged from 62.0 to 89.5 hours; these findings are similar to those reported previously in patients exposed to a higher dose level of 600 mg, where a mean t_1/2_ of approximately 70 hours was observed following multiple administrations of tovorafenib.^12^ A potential limitation of the current study may be that the long terminal phase of the tovorafenib exposure profile and the sampling schedule precluded the inclusion of AUC_0‐inf_ for all groups due to the extrapolated portion of the curve contributing more than 20% to the total AUC value. Comparison of overall exposure between the formulations, and under different prandial states, was evaluated using AUC_0‐last_. The last PK sampling time was 120 hours after dosing. Despite tovorafenib having a long t_1/2_, 120 hours is considered adequate to ensure completion of gastrointestinal transit (approximately 2‐3 days) and absorption of tovorafenib.^29^ Thus, AUC_0‐last_ was sufficient for the comparison of the extent of absorption between the 2 formulations and to assess the food effect. Another limitation of the study is that it was conducted on healthy adult patients; consequently, the findings may not accurately reflect taste perception in infant and pediatric patients,^30^, ^31^, ^32^ especially those who have undergone chemotherapy.^33^ The palatability and acceptability of tovorafenib tablet and PfOS formulations are currently under evaluation as part of the ongoing LOGGIC/FIREFLY‐2 study (NCT05566795) in newly diagnosed *BRAF*‐altered pLGG enrolling pediatric, adolescent, and young adult patients.^15^

Overall, the results from this study indicated that Formulation C (4% strawberry flavor with 0.15% sucralose) was acceptable with good palatability and with comparable exposure to the tablet. Administration of the tovorafenib tablet with a high‐fat meal led to a decrease in the rate of oral absorption; however, this had no impact on the overall extent of absorption of tovorafenib, supporting administration of tovorafenib with or without food.

## Conflicts of Interest

All authors are employees or consultants of Day One Biopharmaceuticals and have received Day One Biopharmaceuticals stock and stock options.

## Funding

This trial was funded by Day One Biopharmaceuticals, Inc.

## Supporting information



Supporting Information

## Data Availability

The data generated in this study are not publicly available due to their being confidential, proprietary company‐owned data but are available upon reasonable request from the corresponding author.
