# Supplementary material for: Taste Profile and Relative Bioavailability of Tovorafenib Powder for Oral Suspension and Food Effect of the Tovorafenib Tablet in Healthy Participants
Source: Clin Pharmacol Drug Dev. 2025 Jun 19;14(9):669–79. doi: 10.1002/cpdd.1558 (PMC12402836; doi:10.1002/cpdd.1558)
Supplement: Supplementary file 1 — Supporting Information [file CPDD-14-669-s001.docx]

**Supplemental Digital Content**

**Figures
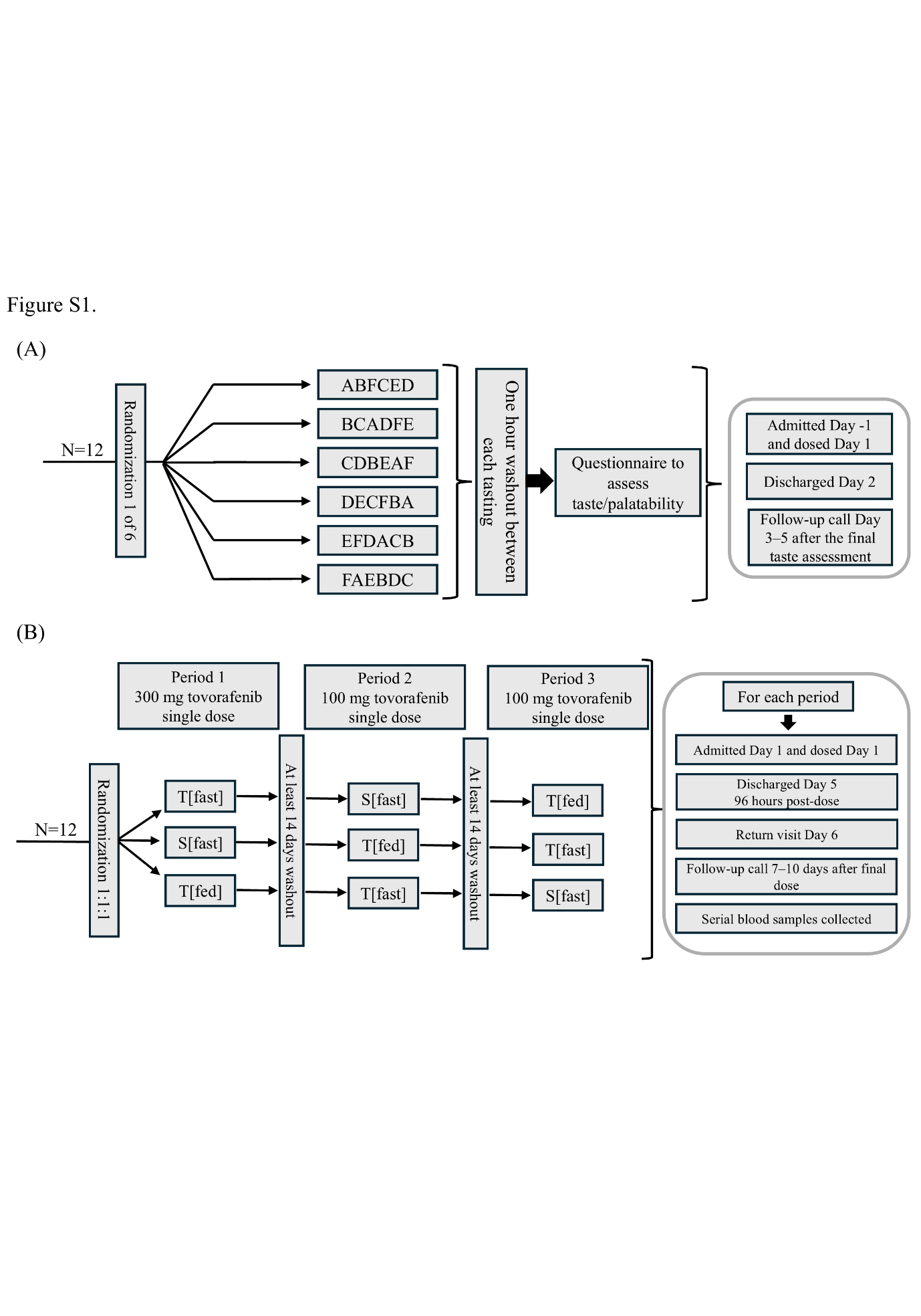
**

**Figure S1.** Study design. (A) Part 1 was designed to evaluate the taste and palatability of 6 different tovorafenib PfOS formulations (A–F). (B) Part 2 was designed to determine the relative bioavailability and evaluate the PK profiles of the preferred tovorafenib preferred PfOS formulation from Part 1 and the tablet formulation and to examine the potential effect of food on the PK of tovorafenib following a single dose of the tablet formulation in healthy participants in the fed state using a randomized, three-period crossover design. Regimens: T[fast]: tovorafenib tablet under fasted; S[fast]: tovorafenib PfOS formulation under fasted; T[fed]: tovorafenib tablet after a high-fat meal.

PfOS, powder for oral suspension; PK, pharmacokinetics.

Figure S2


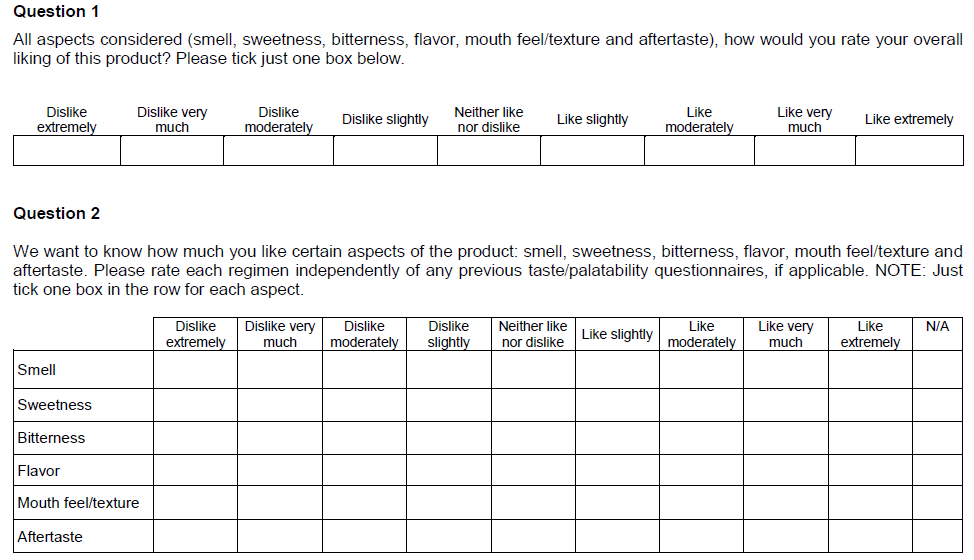
 **Figure S2.** Taste/palatability questionnaire N/A, not applicable.

| **Table S1.** Participant and baseline demographics | | |
| --- | --- | --- |
| Characteristics | Part 1 (N=12) | Part 2 (N=12) |
| Age, years, mean (SD) | 40.3 (4.7) | 42.5 (7.2) |
| Sex, n (%)  Female  Male | 6 (50.0)  6 (50.0) | 6 (50.0)  6 (50.0) |
| BMI, kg/m^2^, mean (SD) | 26.37 (2.47) | 27.58 (2.17) |
| Height, cm, mean (SD) | 166.4 (6.2) | 167.0 (11.2) |
| Weight, kg, mean (SD) | 73.29 (10.54) | 77.36 (12.91) |
| Ethnicity, n (%)  Hispanic or Latino  Not Hispanic or Latino | 11 (91.7)  1 (8.3) | 12 (100)  - |
| Race, n (%)  White  Black or African American | 11 (91.7)  1 (8.3) | 10 (83.3)  2 (16.7) |
| BMI, body mass index; IQR, interquartile range; SD, standard deviation. | | |

| **Table S2.** Part 2 **s**ummary of adverse events in 100 mg and 300 mg tovorafenib | | | | | | | | | | | | |
| --- | --- | --- | --- | --- | --- | --- | --- | --- | --- | --- | --- | --- |
|  | 100 mg dose | | | | | | 300 mg dose | | | | | |
|  | Regimen | | | | | | Regimen | | | | | |
|  | T[fast]  N=7 | | S[fast]  N=8 | | T[fed]  N=8 | | T[fast]  N=4 | | S[fast]  N=4 | | T[fed]  N=4 | |
|  | n (%) | N | n (%) | N | n (%) | N | n (%) | N | n (%) | N | n (%) | N |
| TEAEs | 5 (71.4) | 11 | 5 (62.5) | 8 | 6 (75.0) | 10 | 4 (100) | 18 | 4 (100) | 11 | 4 (100) | 13 |
| Severe TEAEs | 0 (0.00) | 0 | 0 (0.00) | 0 | 0 (0.00) | 0 | 2 (50.0) | 2 | 0 (0.00) | 0 | 2 (50.0) | 2 |
| ADRs | 4 (100) | 7 | 4 (50.0) | 6 | 5 (62.5) | 7 | 4 (100) | 18 | 4 (100) | 11 | 4 (100) | 11 |
| Serious TEAEs | 0 | 0 | 0 (0.00) | 0 | 0 (0.00) | 0 | 0 (0.00) | 0 | 0 (0.00) | 0 | 0 (0.00) | 0 |
| TEAEs leading to study drug withdraw | 0 | 0 | 0 (0.00) | 0 | 0 (0.00) | 0 | 0 (0.00) | 0 | 0 (0.00) | 0 | 0 (0.00) | 0 |
| ADR, adverse drug reaction; n, the number of participants reporting at least one event; N, the total number of events; S[fast], PfOS under fasted; TEAE, treatment-emergent adverse event; T[fast], tablet under fasted; T[fed], tablet after a high-fat meal. | | | | | | | | | | | | |
